# Mouse Embryonic Stem Cells Inhibit Murine Cytomegalovirus Infection through a Multi-Step Process

**DOI:** 10.1371/journal.pone.0017492

**Published:** 2011-03-02

**Authors:** Hideya Kawasaki, Isao Kosugi, Yoshifumi Arai, Toshihide Iwashita, Yoshihiro Tsutsui

**Affiliations:** 1 Department of Second Pathology, Hamamatsu University School of Medicine, Hamamatsu, Japan; 2 Faculty of Health Science, Hamamatsu University, Hamamatsu, Japan; University of Bristol, United Kingdom

## Abstract

In humans, cytomegalovirus (CMV) is the most significant infectious cause of intrauterine infections that cause congenital anomalies of the central nervous system. Currently, it is not known how this process is affected by the timing of infection and the susceptibility of early-gestational-period cells. Embryonic stem (ES) cells are more resistant to CMV than most other cell types, although the mechanism responsible for this resistance is not well understood. Using a plaque assay and evaluation of immediate-early 1 mRNA and protein expression, we found that mouse ES cells were resistant to murine CMV (MCMV) at the point of transcription. In ES cells infected with MCMV, treatment with forskolin and trichostatin A did not confer full permissiveness to MCMV. In ES cultures infected with elongation factor-1α (EF-1α) promoter-green fluorescent protein (GFP) recombinant MCMV at a multiplicity of infection of 10, less than 5% of cells were GFP-positive, despite the fact that ES cells have relatively high EF-1α promoter activity. Quantitative PCR analysis of the MCMV genome showed that ES cells allow approximately 20-fold less MCMV DNA to enter the nucleus than mouse embryonic fibroblasts (MEFs) do, and that this inhibition occurs in a multi-step manner. *In situ* hybridization revealed that ES cell nuclei have significantly less MCMV DNA than MEF nuclei. This appears to be facilitated by the fact that ES cells express less heparan sulfate, β1 integrin, and vimentin, and have fewer nuclear pores, than MEF. This may reduce the ability of MCMV to attach to and enter through the cellular membrane, translocate to the nucleus, and cross the nuclear membrane in pluripotent stem cells (ES/induced pluripotent stem cells). The results presented here provide perspective on the relationship between CMV susceptibility and cell differentiation.

## Introduction

In humans, cytomegalovirus (CMV), a member of the herpes virus family, is the most significant infectious source of intrauterine infections that cause congenital anomalies. Intrauterine infection with human cytomegalovirus (HCMV) is thought to be responsible for a variety of abnormalities, depending on the timing of fetal infection, infectious route, and virulence of the virus [1]. Differential susceptibility of certain early embryonic cells to HCMV infection may cause abnormal embryogenesis or organogenesis, resulting in central nervous system defects. Previous studies have demonstrated altered susceptibility to CMV infection among different cell types, including various types of stem/progenitor cells [2], [3], [4], [5], [6], [7]. This can cause abnormal embryogenesis and/or organogenesis, which, in turn, results in congenital anomalies [8].

Studies of human subjects have obvious limitations, but CMVs exhibit strict species specificity, and HCMV therefore cannot be studied directly in any laboratory animal. Thus, general CMV pathogenesis has been examined in mice, using murine CMV (MCMV) [9], [10], and in guinea pigs, using guinea pig CMV[11]. Interestingly, mouse embryos injected with MCMV-infected blastocysts do not express viral genes, suggesting that they are not susceptible to MCMV [12]. Further, mouse embryonic stem (ES) cells are non-permissive to MCMV infection, and the MCMV immediate-early (IE) promoter is not activated in ES cells from transgenic mice [4]. Human NTera2/D1 embryonic carcinoma cells (NT2) are a useful model in which to study the regulatory mechanisms behind major immediate-early (MIE) enhancer/promoter silencing during quiescent HCMV infection [5], [13], [14]. This is because HCMV replication is prevented in embryonic NT2 cells, where viral MIE gene expression is blocked, but not in differentiated cells [5], [13], [15], [16]. Trichostatin A (TSA), an inhibitor of histone deacetylases, brings about MIE enhancer/promoter reactivation in quiescently infected NT2 [17], independent of cellular differentiation [18]. Treatment with TSA disrupts heterochromatin nucleation at the MIE enhancer/promoter [18], a process akin to the chromatin disruption that accompanies HCMV reactivation in endogenously-infected dendritic cells [6]. Stimulation of the cyclic AMP (cAMP)/protein kinase A signaling pathway drives cAMP response element (CRE)-dependent MIE enhancer/promoter activation in quiescently infected NT2 cells, thus exposing a potential mode of regulating HCMV reactivation [19]. Whether these mechanisms also regulate CMV infection in ES cells remains unknown.

There are multiple stages to the CMV infection process. First, the virus attaches to the (mammalian) host cell surface via interaction between an envelope component and a cellular molecule that serves as a receptor. After attachment, the virus must cross the plasma membrane during a phase of its life cycle known as penetration. The viral particle is very large, and no infectious core particle has ever been observed in the nucleus; this suggests that the virus is disassembled prior to nuclear entry. Finally, viral DNA, or a DNA-protein complex, enters the nucleus. MCMV genes are expressed in 3 sequential phases: immediate-early, early, and late [20].

In this work, we investigated the susceptibility of mouse ES cells to MCMV by comparing each step of the infection process (e.g., attachment, entry, trafficking, nuclear entry, and promoter activity) between the most susceptible murine cells, mouse embryonic fibroblasts (MEFs) and ES cells. We found that ES cell susceptibility is inhibited in a multi-step manner. Additionally, we have shown that induced pluripotent stem (iPS) cells, like ES cells, are MCMV-resistant. We describe novel characteristics of pluripotent stem cells (ES or iPS) and their response to CMV infection.

## Results

### Establishment of ES cell lines

**Figure 1:**
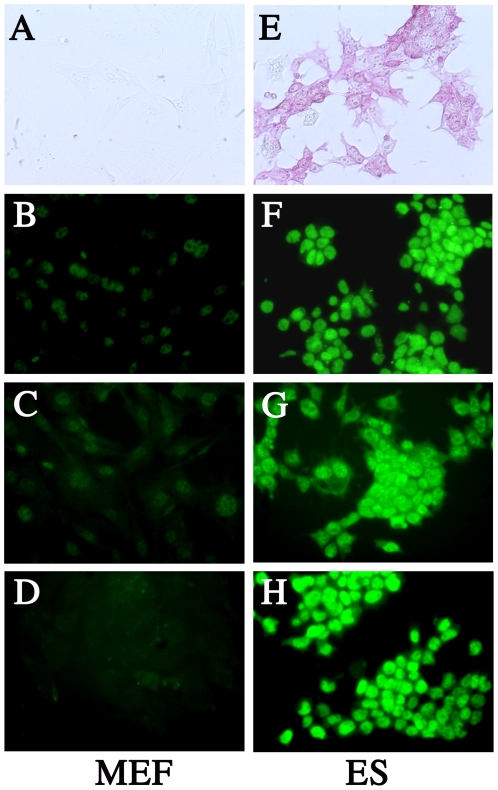
Establishment of ES cell lines. Unlike MEF (A-D), ES cells had alkaline phosphatase activity (E) and expressed SOX2 (F), Nanog2 (G), and OCT3/4 (H).

C57BL/6 blastocysts obtained by mating MCMV IE promoter-*lacZ* transgenic mice with C57BL/6 mice were cultured in ES/fetal calf serum (FCS) medium. Eight ES cell lines were established after the successful growth of inner cell masses. Using previously described methods, we confirmed that the cell lines contained a portion of the MCMV IE promoter-*lacZ* (407 bp) [21]. One of these cell lines was used for all subsequent experiments, along with an IE promoter-negative cell line. Pluripotent differentiation ability was confirmed in the ES T-1 cells by intraperitoneally injecting them into young adult C57BL/6 mice and verifying the formation of teratomas. The resulting tumor masses consisted of both undifferentiated and differentiated cells. The differentiated cells (e.g., epithelial cells, neural cells, chondrocytes, striated muscle cells, and digestive gland cells) were observed in all 3 embryonic germ layers, as previously described [4]. Cell marker activity was compared between ES cells and mouse embryonic fibroblasts (MEFs). Unlike MEFs (Figure 1A–D), ES cells had alkaline phosphatase activity (Figure 1E) and expressed SOX2 (Figure 1F), Nanog2 (Figure 1G), and OCT3/4 (Figure 1H).

### ES cell resistance to MCMV infection

**Figure 2:**
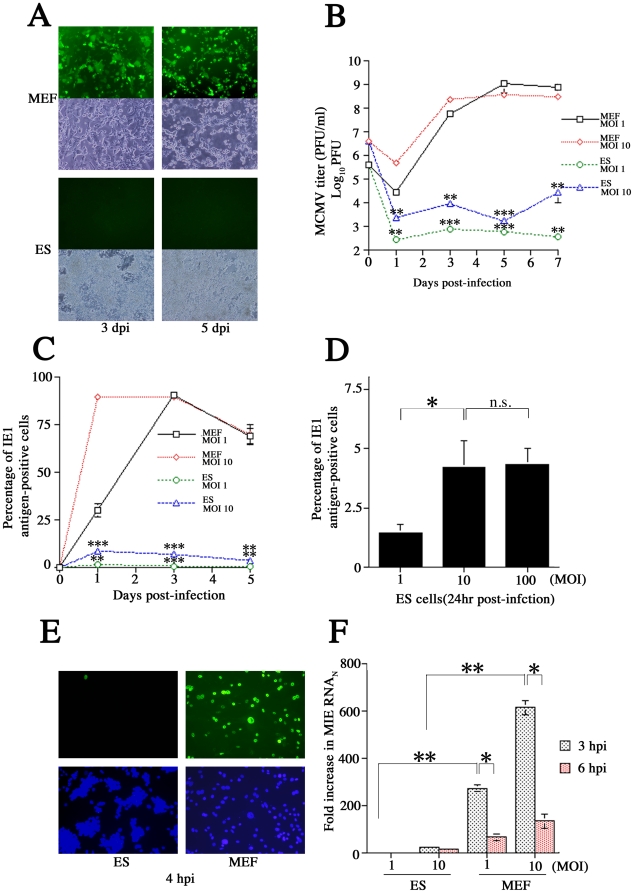
Resistance to MCMV in ES cells. (A) Differences in GFP expression in MEF and ES cells at 3 and 5 dpi with MCMV (RM4503). (B) The time course of MCMV virus (Smith strain) titer in MEF and ES cells infected at MOIs of 1 and 10. Each *P*-value was calculated between MEF and ES at an MOI of 1 or 10 of the same days post-infection (dpi). (C) Differences in the proportions of IE1-positive cells in MEF and ES cultures infected at MOIs of 1 and 10. Each *P*-value was calculated between MEF and ES at an MOI of 1 or 10 of the same dpi. (D) Comparison of infection levels at different MOI (1, 10, 100) at 24 hpi. ES cells infected at MOIs of 10 and 100 had similar proportions of IE1-positive cells (*P*>0.05: n.s.). (E) IE-positive cells during the immediate-early phase of infection in MEF and ES cells at 4 hpi. (F) MIE RNA levels in MEF and ES cells at 3 and 6 hpi. All presented experiments were performed at least 3 times, and data are given as the mean±SD. * *P*<0.05, ** *P*<0.01, *** *P*<0.001, n.s.: not significant, *t*-test.

We evaluated the susceptibility of ES cells to MCMV by comparing the infection rate of ES cells and MEFs after exposure to MCMV. Both cell types were infected with MCMV (RM4503, derived from the K181 strain) at a multiplicity of infection (MOI) of 1 (determined on MEF). The infected cells first exhibited green fluorescence protein (GFP) fluorescence at 3 days post-infection (dpi), with the intensity increasing by 5 dpi in MEF (Figure 2A). The cytopathic effect, which indicates the beginning of lytic cell death, was observed in MEF at 3 dpi, whereas ES cells were rarely GFP-positive and exhibited little cytopathic effect even after 5 dpi (Figure 2A). We evaluated viral titer over time by comparing values in MEF and ES cells at MOIs of both 1 and 10 (Figure 2B). At 5 dpi, MEFs yielded 10^4^ to 10^5^ times more progeny virus than ES cells at both MOIs (*P*<0.001). Flow cytometry, using the IE1 antigen-specific monoclonal antibody (mAb) N2 [22], was used to evaluate the proportions of cells infected at each time point. At 3 dpi, 90.6% and 89.3% of MEF were IE1-positive when infected at MOIs of 1 and 10, respectively. In ES cells infected at the same MOIs, only 0.73% and 6.87% of cells were IE1-positive by day 3 (*P*<0.001) (Figure 2C). This suggests that ES cells have considerable resistance to CMV.

To analyze the effect of MCMV concentration on ES cells, the numbers of IE1-positive cells were measured after infection at different MOIs (1, 10, 100) at 24 hours post-infection (hpi). There were more IE1-positive cells after infection at an MOI of 10 than at an MOI of 1. However, the proportions of IE1-positive cells were similar after infection at MOIs of 10 and 100 (*P*>0.05: not significant). Thus, while susceptibility to MCMV seems to be concentration-dependent, ES cells reach MCMV saturation at an MOI of 10 (Figure 2D). Immunocytochemistry showed that, at 4 hpi at an MOI of 10, MEF cultures had many IE1-positive cells, while ES cultures had almost none (Figure 2E). This strongly suggests that the 2 cell types differ in susceptibility prior to the IE phase.

To analyze when ES inhibits MCMV infections (e.g., pre- or post-transcription), real-time RT-PCR was used to quantify the MIE RNA produced by the MCMV (Smith strain) at 3 and 6 hpi after infection at MOIs of 1 and 10. Each sample was normalized to concomitantly measured 18S rRNA values. At 3 hpi at MOIs of 1 and 10, MIE RNA production was 218.2 and 27.9 times higher, respectively, in MEFs than in ES cells (*P*<0.01). MIE RNA production in MEFs was significantly lower at 6 hpi than at 3 hpi (*P*<0.05) (Figure 2F). This suggests that the 2 cell types differ in susceptibility at the point of transcription.

### Transfected/integrated MCMV IE promoter activity

**Figure 3:**
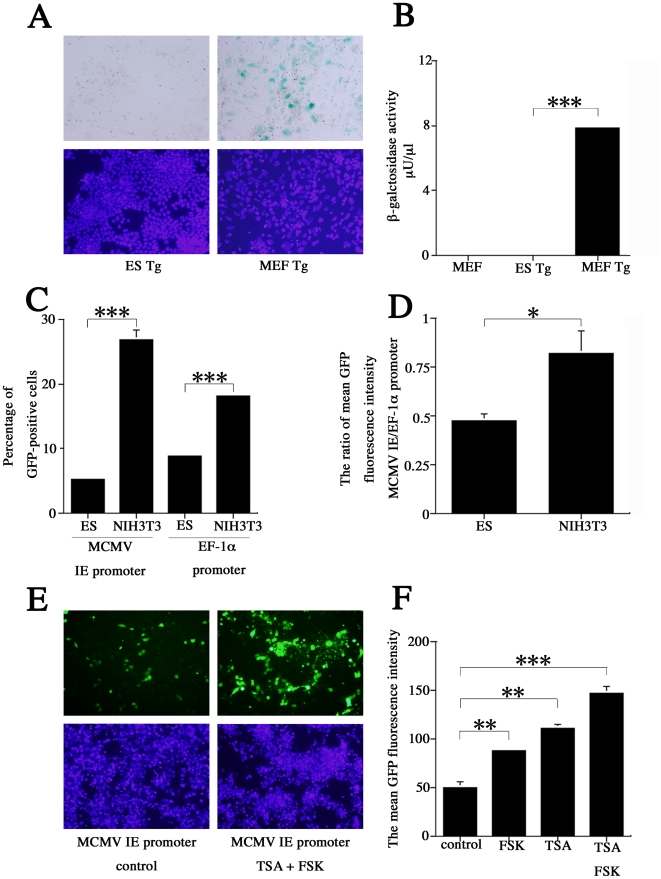
Comparison of transfected/integrated MCMV IE promoter activity in MEF and ES cells. (A) Results of the galactosidase enzyme assay on transgenic ES cells and MEF. β–gal positive cells (blue). (B) β–gal activity in wild-type MEF and transgenic ES cells and MEF. Activity could only be detected in transgenic MEF, at 7.9 µU/µL. (*P*<0.001) (C, D) Detection of promoter activity by flow cytometric analysis of GFP reporter expression. (C) The percentage of GFP positive cells with MCMV IE promoter activity and EF-1α promoter activity. (D) MCMV IE promoter activity after normalization with EGFP expression values under the control of the EF-1α promoter. There was a significant difference between ES and NIH3T3 after normalization (*P*<0.05). (E, F) Examination of the influence of histone modification and stimulation of the cAMP pathway on the transfected MCMV IE promoter in ES cells. (E) Co-treatment with FSK and TSA drastically increased the number of GFP-positive cells. (F) FSK and TSA each increase GFP fluorescence activity when administered individually (FSK: *P*<0.01, TSA: *P*<0.01), but the strongest response was seen when they were administered together (*P*<0.001). All presented experiments were performed at least 3 times, and data are given as the mean±SD. * *P*<0.05, ** *P*<0.01, *** *P*<0.001, *t-*test.

Expression of the IE gene is highly dependent on cellular transcription factors that bind to the DNA, including the enhancer sequence, of the IE promoter [23]. We examined activation of the integrated MCMV IE promoter in ES cells and MEFs derived from MCMV IE promoter-*lacZ* transgenic mice. Transgenic ES cell cultures contained no 5-bromo-4-chloro-3-indoyl-β-D-galactopyranoside (X-Gal)-positive cells, but the MEF cultures did (Figure 3A). A galactosidase enzyme assay was used to measure β-galactosidase (β-gal) activity. In ES cells, as in the negative controls, no activity was detected; MEFs showed 7.9 µU/µL β-gal activity (*P*<0.001) (Figure 3B). A similar pattern was previously reported elsewhere [4], and these results are consistent with our observations of infectious activity in both cell types.

A growing number of reports indicate that transient transfection promoters and integrated promoters elicit different expression patterns [24]. Therefore, we also investigated MCMV IE promoter activity using the transient transfection method. As MEFs are not easily transfected, we used immortalized mouse embryonic fibroblast cells (NIH3T3) to achieve efficient transfection. Attractene Transfection Reagent was used to transfect NIH3T3 and ES cells, which were subjected to flow cytometry 24 h post-transfection. Enhanced GFP (EGFP) expression under control of the elongation factor-1α (EF-1α)/HTLV composite promoter was also analyzed, and these values were used to normalize data collected from ES and NIH3T3 cells. After transfection with the MCMV IE promoter, NIH3T3 cultures had a much higher percentage than ES cultures of GFP-positive cells (26.8% vs. 5.2%) (*P*<0.001) (Figure 3C). After transfection with the EF-1α promoter, 18.2% of NIH3T3 and 8.8% of ES cells were GFP-positive (*P*<0.001). Once MCMV IE promoter activity was normalized using EF-1α/HTLV values, ES cells were found to have significantly less (1.7-fold) activity than NIH3T3 cells (*P*<0.05) (Figure 3D). These results confirmed previous reports that the MCMV IE promoter plays an important role in MCMV infection in ES cells [4].

There are a variety of mechanisms whereby ES cells may silence the MCMV IE promoter. One of these involves chromatin structure, which plays an important role in regulating gene expression. For instance, TSA, a histone deacetylase inhibitor, reactivates HCMV major immediate-early regulatory region-dependent transcription [17], while forskolin (FSK), an adenylyl cyclase activator, greatly alleviates MIE enhancer/promoter silencing by stimulating the cAMP pathway in quiescently infected NT2 cells [19]. Therefore, we examined the influence of histone modification and stimulation of the cAMP pathway on the transfected MCMV IE promoter in ES cells. FSK (50 µM) and TSA (100 ng/mL) were added to the culture 4 hours after ES cells were transfected with a plasmid containing the MCMV IE promoter. This led to a significant increase in the number of GFP-positive cells and the intensity of GFP fluorescence, compared to cultures treated with FSK/TSA (Figure 3E). Thus, FSK and TSA synergistically activate the MCMV IE promoter in ES cells (*P*<0.001) (Figure 3F).

### Effects of FSK and TSA in MCMV-infected ES cells

**Figure 4:**
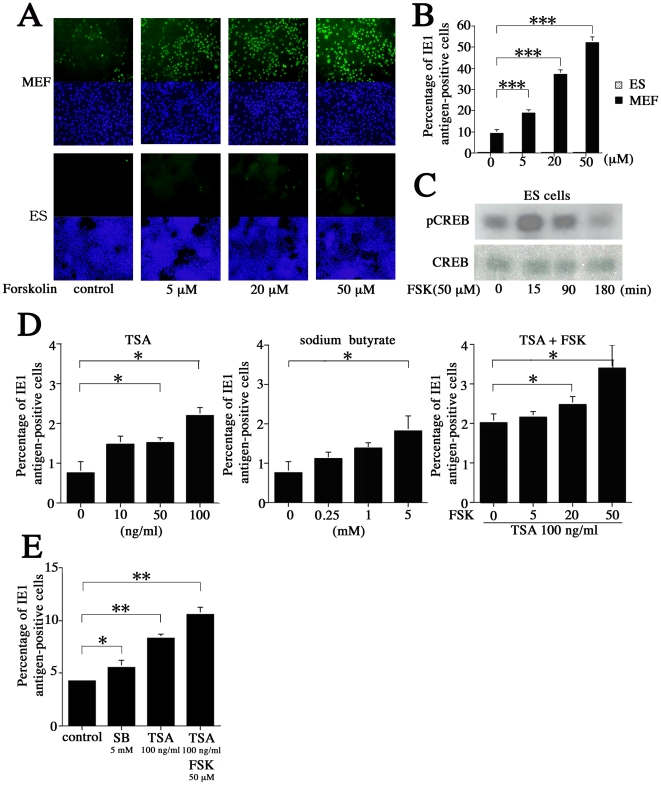
Response of the MCMV lytic cycle to treatment with FSK and TSA. Both (A) immunocytochemical and (B) flow cytometric analyses showed that treatment with FSK increases the proportion of IE1-positive cells in MEF cultures in a concentration-dependent manner (*P*<0.001), while only slight increases were seen in ES cells. (C) Western blot evaluating levels of phosphor-Ser 133 CREB after treatment with FSK. (D) Percentage of IE1 antigen-positive cells at 24 hpi in ES cells infected with MCMV at an MOI of 1 prior to treatment with TSA (left), sodium butylate (SB; middle), and a combination of TSA (100 ng/mL) and FSK (0–50 µM; right). (E) Percentage of IE1-positive cells in ES cells infected with MCMV at an MOI of 10 prior to treatment with SB (5 mM), TSA (100 ng/mL), or a combination of TSA (100 ng/mL) and FSK (50 µM). All presented experiments were performed at least 3 times, and data are given as the mean±SD. * *P*<0.05. **P<0.01, *** *P*<0.001, *t-*test.

As previously described, stimulating the cAMP pathway with FSK greatly alleviates MIE enhancer/promoter silencing in quiescently infected NT2 cells [19]. Thus, we examined whether cAMP stimulation influences ES cell susceptibility to MCMV. As a control, MEFs were deprived of serum for 16 h prior to infection. ES cells and MEFs were infected with MCMV at an MOI of 1. After 2 hours, 5, 20, or 50 µM FSK was added to each culture (Figure 4A). Both immunocytochemistry and flow cytometry indicated that FSK increased the percentage of IE1-positive cells in MEF cultures in a concentration-dependent manner (from 9.16% in untreated cultures to 52.1% in cultures treated with 50 µM FSK) (*P*<0.001) (Figures 4A and 4B). In ES cells, on the other hand, there was very little response to FSK stimulation (from 0.64% IE-positive cells in untreated cultures to 0.80% in cultures treated with 50 µM FSK) (Figures 4A and 4B).

FSK stimulation activates CRE-dependent transcription through the signaling-mediated phosphorylation of Ser133 in the cAMP response element binding protein (CREB) [25], [26]. By measuring levels of phosphor-Ser133 CREB using a western blot assay, it was possible to determine whether ES cells responded to FSK stimulation. After treatment with FSK, CREB was maximally phosphorylated at 15 min, but levels were reduced by 90 minutes, before returning to pre-stimulus levels by 180 minutes post-stimulus (Figure 4C).

ES cultures infected with MCMV at an MOI of 1 were treated with 4 different concentrations of TSA (Figure 4D, left) and sodium butylate (SB) (Figure 4D, middle). The percentage of IE1-positive cells was measured at 24 hpi and increased in concentration-dependent response to TSA and SB (*P*<0.05). Furthermore, when 100 ng/mL TSA and 50 µM FSK were added simultaneously to ES cells infected with MCMV at an MOI of 1, they worked synergistically to increase the percentage of IE1-positive cells to 3.4% (*P*<0.05) (Figure 4D, right). ES cultures infected with MCMV at an MOI of 10 were then treated with SB (5 mM) or TSA (100 ng/mL), leading to 5.2% (*P*<0.05) and 7.7% (*P*<0.01) increases in IE1-positive cells, respectively. However, we observed the strongest response when 100 ng/mL TSA and 50 µM FSK were added simultaneously; their synergistic effects led to an 11.5% increase in IE1-positive cells (*P*<0.01) (Figure 4E). TSA and FSK treatment did not confer full permissiveness to MCMV in ES cells, such that they behaved like MEFs. This raised the possibility that ES cells might have other MCMV resistance mechanisms.

### Effects of the elongation factor-1α promoter in ES cells infected with recombinant MCMV

**Figure 5:**
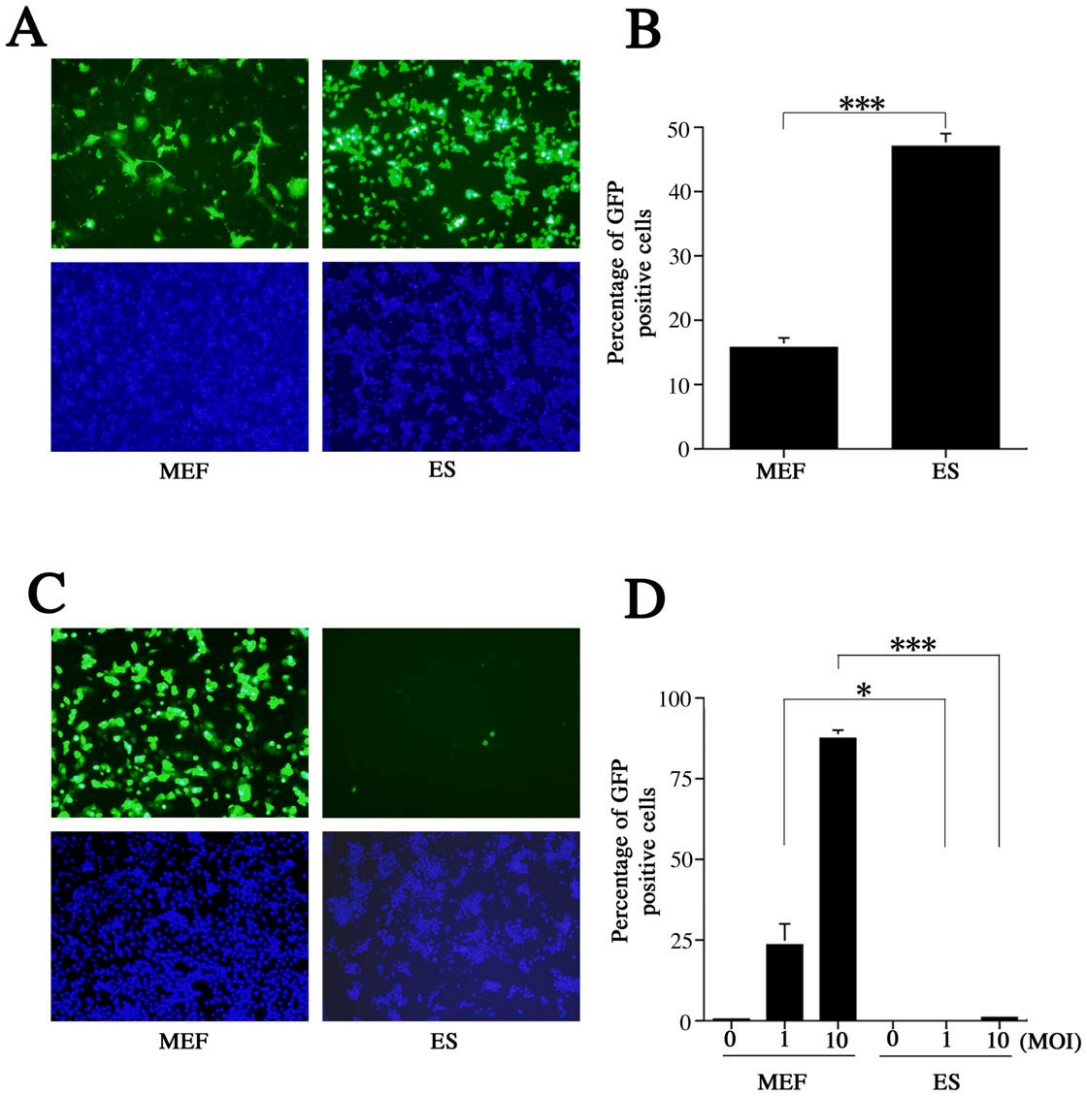
Effects of the elongation factor-1α promoter in ES cells infected with recombinant MCMV. (A) GFP-positive cells in ES and MEF cultures after transfection via electroporation (green: GFP; blue: DAPI) at 24 hours post-transfection. (B) ES cultures had nearly 3 times as many GFP-positive cells (46.9%) as MEF cultures (15.8%) at 24 hours post-transfection (*P*<0.001). (C) Recombinant MCMV was constructed to express an EGFP gene insert under control of EF-1α promoter. GFP positive cells were barely detectable in ES cells at 24 hpi after infection at an MOI of 10 (green: GFP, blue: DAPI). (D) The proportion of GFP-positive cells was significantly higher in MEFs than in ES cells infected at MOIs of 1 (*P*<0.05) and 10 (*P*<0.001). All presented experiments were performed at least 3 times, and data are given as the mean±SD. * *P*<0.05, **P<0.01, *** *P*<0.001, *t-*test.

Using the adenovirus vector transfection system, Kawabata et al. found that the EF-1α promoter is more efficient than the Rous sarcoma virus promoter, the CMV promoter, and the β-actin promoter/CMV enhancer, in mouse ES cells [27]. After confirming EF-1α promoter activity in ES cells (Figure 3C), we used the Amaxa electroporation transfection system to improve transfection efficiency in ES cells and MEFs. GFP-positive cells under the control of the EF-1α promoter accounted for as much as 46.9% of ES cultures, while only 15.8% of MEFs were GFP-positive (*P*<0.001) (Figures 5A and 5B). This indicated that ES cells possess the intrinsic transcription factors necessary to activate the EF-1α promoter.

Next, we generated a recombinant virus to express EGFP under control of the EF-1α promoter (EF-1α recombinant MCMV). In MEFs infected with recombinant virus, GFP activity was quite high (Figure 5C) and occurred in an MOI-dependent manner (Figure 5D). In contrast, ES cells were resistant to the recombinant MCMV. Even at an MOI of 10, there were few GFP-positive ES cells (Figure 5C), and the total proportion of GFP-positive cells was much lower in ES than in MEF cultures at MOIs of 1 (*P*<0.05) and 10 (*P*<0.001) (Figure 5D).

### Centrifugation of MCMV onto ES cells enhances infectivity

**Figure 6:**
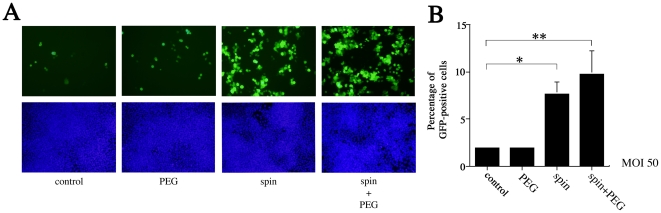
Effects of polyethylene glycol and centrifugation on EF-1α-promoter recombinant MCMV infection in ES cells. (A) GFP-positive cells in ES cultures treated with 44% PEG, centrifugation + PBS, or centrifugation +44% PEG after infection with EF-1α recombinant MCMV at an MOI of 50. (B) Flow cytometry was used to verify the proportion of GFP-positive cells in each of the infection treatments. All presented experiments were performed at least 3 times, and data are given as the mean±SD. * *P*<0.05, ** *P*<0.01, *t-*test.

We used centrifugation and polyethylene glycol (PEG) treatments to determine whether MCMV resistance in ES cells is mediated at the adsorption or entry steps. Centrifugation is thought to enhance the infectivity of HCMV by increasing adsorption [28]. Enveloped viruses reach the cytoplasm by numerous mechanisms, including fusion with the plasma membrane and endocytosis followed by fusion with endosomal membranes [29], [30]. PEG chemically induces fusion by dehydrating the surfaces of juxtaposed membranes [31] and essentially bypasses normal entry processes by inducing fusion with the plasma membrane. In this way, PEG can force the entry of otherwise entry-defective herpesviruses when adsorbed onto the cell surface [32]. ES cells were inoculated with EF-1α recombinant MCMV at an MOI of 50, with or without centrifugation, and then subjected to a brief treatment with 44% PEG. Centrifugation significantly (3.9-fold) increased the proportion of GFP-positive cells (7.6%) in ES cultures (*P*<0.05) (Figure 6A and 6B). PEG treatment without prior centrifugation had little effect on MCMV susceptibility. However, PEG treatment with prior centrifugation increased the proportion of GFP-positive cells more significantly (9.8%) (*P*<0.01) (Figures 6A and 6B). MEF cultures treated with both centrifugation and PEG had 90.0% GFP-positive cells (data not shown). Although it is not clear whether ES cells internalize MCMV via fusion at the plasma membrane or endocytosis, these results suggest that infection was blocked at one or more post-entry stages of infection, and that this blockage could not be overcome with PEG treatment with centrifugation.

### Multi-step inhibitory regulation of MCMV infection

**Figure 7:**
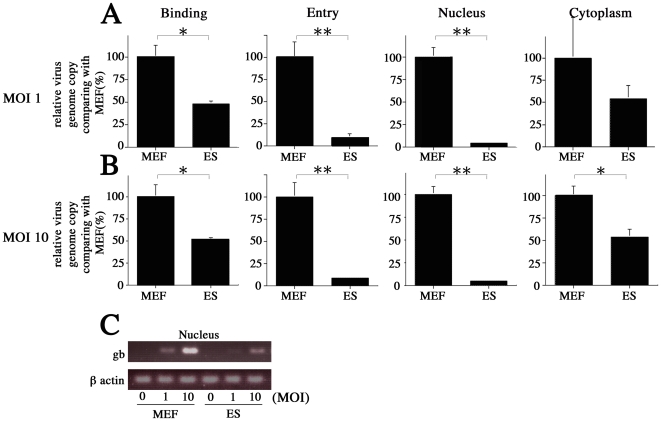
Comparison of MCMV infection inhibition at each stage of the infection process in MEF and ES cells. The amount of MCMV DNA was quantified at the binding and entry stages and in the nucleus and cytoplasm of MEF and ES cells infected with MCMV at MOIs of (A) 1 and (B) 10. (C) Results from semi-quantitative PCR used to measure the amount of MCMV DNA in the nuclei of MEF and ES cells. All presented experiments were performed at least 3 times, and data are given as the mean±SD. * *P*<0.05, ** *P*<0.01, *t-*test.

We next compared the amounts of MCMV DNA at each step of the infection process (binding, cell entry, nucleus entry) in ES cells (values standardized by β-actin) and MEFs at 2 different MOIs. MCMV was absorbed into ES and MEF at 4°C for 1 hour. The cells were washed 3 times with cold PBS. Viral DNA was extracted from a portion of these cells so the amount of MCMV during the binding process could be measured. The remaining cells were incubated at 37°C for 2 h so the virus could undergo cell entry. Virions that did not penetrate cells were removed by EDTA-trypsin, while the internalized viral DNA was quantified by real-time PCR. Finally, we collected the nuclear and cytoplasmic fractions using previously-described protocols [33]. Viral DNA was extracted from each fraction to determine the numbers of MCMV particles associated with nuclear entry. MCMV DNA levels in MEFs and ES cells were standardized to β actin. Cumulatively, our results indicate that ES cells exhibit multi-step inhibition. In ES cells infected at MOIs of 1 and 10, MCMV DNA levels at the binding stage were 53.2% and 51.3% lower, respectively, than they were in MEFs (*P*<0.05) (Figures 7A and 7B). There was an even greater discrepancy between ES cells and MEFs at the entry point, when MCMV DNA levels were 91.2% and 90% lower in ES cells infected at MOIs of 1 and 10, respectively (*P*<0.01) (Figures 7A and 7B). At the nuclear entry step, ES cells infected at MOIs of 1 and 10 had 95.1% and 95.0% less MCMV DNA, respectively, than MEFs (*P*<0.01). Finally, the MEF cytoplasm contained about twice as much remnant MCMV DNA as the ES cell cytoplasm, at MOIs of both 1 and 10 (Figure 7A and B). Semi-quantitative PCR also showed that ES cells contained significantly less MCMV DNA in their nuclei than MEFs did, at MOIs of both 1 and 10 (Figure 7C).

### Visualization of the MCMV genome in ES cells using *in situ* hybridization

**Figure 8:**
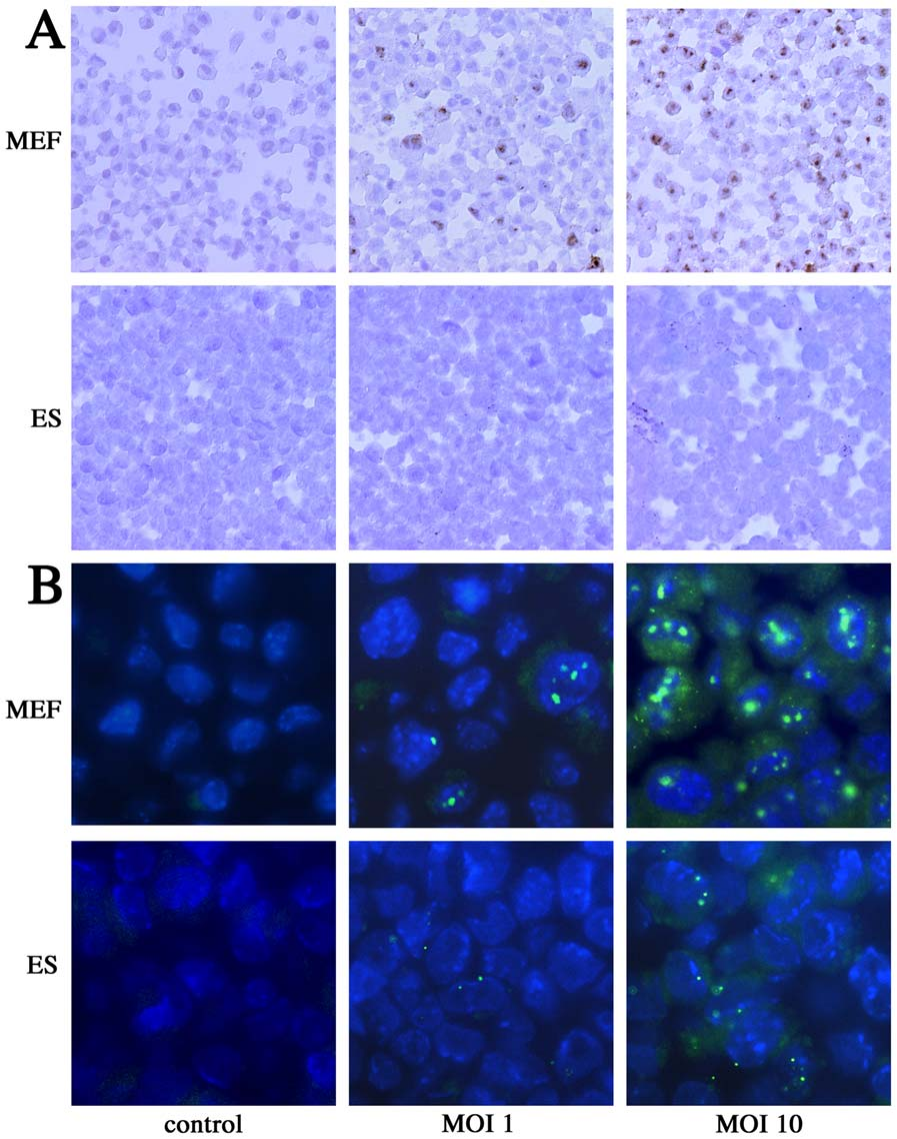
Visualization of the MCMV genome by *in situ* hybridization in MEF and ES cells. To confirm the results of the DNA quantification experiments, *in situ* hybridization was used to visualize the MCMV genome in MEF and ES cells infected with MCMV at MOIs of 1 and 10. The DNA was visualized by (A) immunostaining with DAB (brown) or by a (B) GFP signal (green).

We used *in situ* hybridization (ISH) to confirm that ES cells contained less MCMV DNA at each stage of the infection process (Figure 7). The probe used for DNA *in situ* hybridization was made from a BAC library containing the MCMV DNA genome, pSM3fr, as previously described [34]. MEFs and ES cells were infected with MCMV at MOIs of 1 and 10. At 2 hpi, each cell type was fixed with 4% paraformaldehyde and paraffin-sectioned. MCMV DNA was immunostained with peroxidized GFP antibody and visualized either with DAB staining (brown; Figure 8A) or by the GFP signal directly conjugated with probes (green; Figure 8B). Neither signal was detected in the control (non-infection) sections (Figures 8A and 8B, left panel). As the infection concentration increased, MCMV DNA signaling increased in both ES cells and MEFs (Figures 8A and 8B, middle and right panels). However, there was significantly less signaling in ES cells than in MEFs, and although most MEF signals were localized to the nucleus, this was rarely the case in ES cells. Thus, the ISH results confirmed the findings of our real-time PCR analyses (Figure 7).

### Resistance to MCMV in iPS

Matsukage et al. reported that ES cells became more susceptible to MCMV after undergoing differentiation [4]. However, the susceptibility of CMV to iPS has not yet been addressed. To create dedifferentiated cells, Takahashi et al. reported that 4 factors (OCT4, SOX2, c-Myc, Klf-4) are sufficient to reprogram somatic cells (fibroblasts) to pluripotent stem cells that exhibit the essential characteristics of ES cells [35].

**Figure 9:**
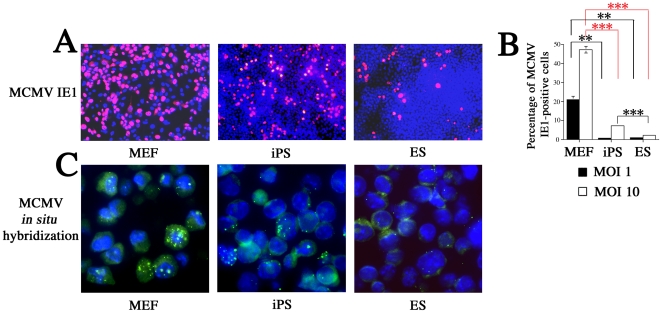
Comparison of MCMV resistance in MEF, iPS, and ES cells. (A) Results from immunocytochemical analyses visualizing MCMV IE1-positive cells (red) in the 3 cell cultures after infection with MCMV at an MOI of 10. (B) Flow cytometry was used to confirm that iPS and ES both had lower numbers of IE1-positive cells than MEF, when infected at MOIs of 1 (*P*<0.01) and 10 (*P*<0.001). At an MOI of 10, iPS cells showed higher expression than ES cells of IE1 (7.27% vs. 2.17%) (*P*<0.001). (C) Results from *in situ* hybridization showing that MCMV signaling in all 3 cultures was proportional to the amount of IE1-positive cell expression. All presented experiments were performed at least 3 times, and data are given as the mean±SD. ** *P*<0.01, *** *P*<0.001, *t-*test.

We acquired a mouse iPS cell line established with Oct3/4, Sox2, Klf4, and c-myc (RIKEN BioResource Center). The GFP gene was knocked-in under the Nanog promoter, allowing detection of GFP in undifferentiated cells [36]. When infected at an MOI of 1, iPS and ES cultures had similar levels of IE1-positive cells (0.85% and 1.02%, respectively), both of which were lower than the levels observed in MEFs (21.1%) (*P*<0.01) (Figure 9B). When infected at an MOI of 10, iPS cultures had a higher proportion of IE1-positive cells (7.27%) than did ES cultures (2.17%) (*P*<0.001), both of which were significantly lower than the levels observed in MEFs (47.2%) (*P*<0.001) (Figures 9A and 9B). Interestingly, the majority of IE1- and Nanog-positive cells in iPS cultures co-localized (data not shown), indicating that the susceptibility of iPS is independent of differentiation.

After 3 hpi, we performed ISH to observe MCMV DNA signals in MEF, iPS, and ES cells. ISH signaling peaked in MEF cultures, was intermediate in iPS cultures, and was weakest in ES cultures (Figure 9C), confirming the results of our immunocytochemical analyses (Figure 9A). Thus, while iPS and ES cells are both more resistant to MCMV than MEFs, iPS appears to be more susceptible than ES cells to MCMV.

### Analysis of factors thought to confer resistance to MCMV in pluripotent cells

**Figure 10:**
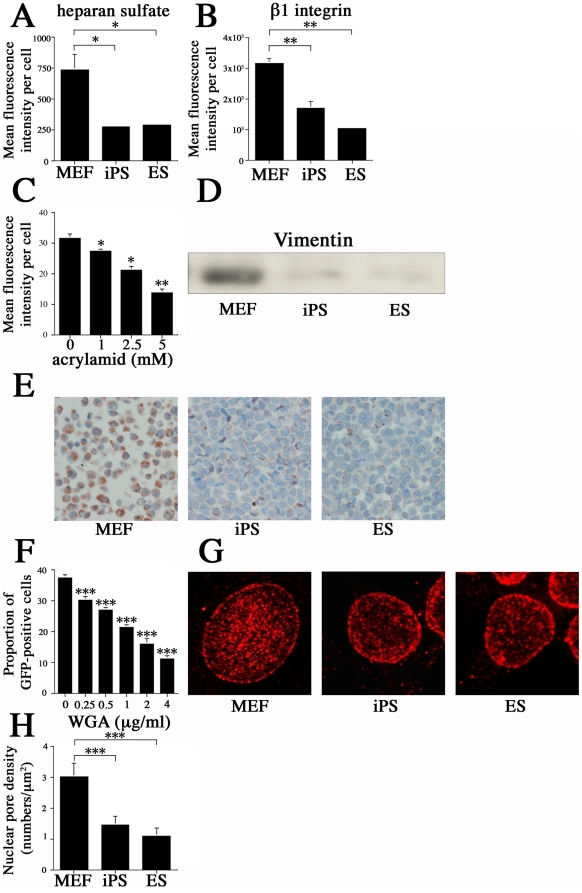
Analysis of factors thought to confer resistance to MCMV in pluripotent cells. ES cells, iPS cells, and MEF were immunostained with (A) heparan sulfate antibody and (B) β1 integrin antibody. Flow cytometry showed that MEFs have 2.6 times more fluorescence intensity per cell than iPS and ES cells after staining with heparan sulfate antibody (*P*<0.05), and 1.8 and 3.0 times more fluorescence intensity per cell than iPS and ES cells, respectively, after staining with β1 integrin antibody (*P*<0.01). (C) Effects of acrylamide treatment on MCMV susceptibility shown as the average mean fluorescence intensity per cell at 4 hpi in MEF infected with EF recombinant MCMV (*P*<0.05, *P*<0.01 vs control). (D) Western blot showing vimentin expression in MEF, iPS, and ES cells. (E) Immunocytochemical analyses of vimentin signals in MEF, iPS, and ES cells. (F) Proportion of GFP-positive cells in MEF cultures infected with MCMV (EF-1α recombinant MCMV) at an MOI of 10 and treated with WGA to block nuclear pores (*P*<0.001 vs control). (G) Confocal images reveal the nuclear pore density in MEF, iPS, and ES cells. (H) Densitometric analysis showed that the density of nuclear pores was higher in MEF than in iPS or ES cells (*P*<0.001). All presented experiments were performed at least 3 times, and data are given as the mean±SD. * *P*<0.05, ** *P*<0.01, *** *P*<0.001, *t-*test.

We investigated the role of various factors thought to affect each step of the MCMV infection process. When binding to the cell, the virus engaged cell-surface heparan sulfate (HS), a relatively conserved feature of the herpesvirus entry pathway [37]. Average mean HS fluorescence intensity was 2.6-fold less in both ES cells and iPS than in MEFs (*P*<0.05) (Figure 10A). Additionally, when MCMV was pretreated with heparin prior to infection, infectivity was significantly reduced in MEFs (data not shown). Cells such as NIH3T3, which are treated with the anti-β1 integrin-neutralizing antibody (clone DE9), exhibit significant (> 80%) reductions in MCMV infectivity, suggesting that MCMV primarily uses a β1 integrin-specific entry pathway [38]. The average mean fluorescence intensity of β1 integrin per cell is 1.8-fold lower in iPS and 3.0-fold lower in ES cultures than in MEFs (*P*<0.01) (Figure 10B).

During the initial phase of HCMV infection, the virus requires an intact network of vimentin intermediate filaments, which appear to facilitate capsid trafficking and/or docking to the nuclear envelope [39]. To investigate whether vimentin is also important in MCMV infections, we treated MEFs with acrylamide, which chemically disrupts the vimentin network, prior to infecting them with EF-1α recombinant MCMV at an MOI of 10. At 4 hpi, we measured the average fluorescence intensity in each cell and found that disruption of the vimentin network reduced MCMV entry in a concentration-dependent manner (Figure 10C). Western blotting and immunocytochemical analysis revealed that per-cell expression of vimentin was significantly lower in iPS and ES cells than in MEFs (Figures 10D and 10E).

The precise mechanisms by which CMV enters the nucleus after penetrating the cell are not yet understood, though it is known that it passes through nuclear pores [20]. Importin β mediates the process by which the herpes simplex virus (HSV) capsid docks at the nuclear pore complex (NPC), after which the viral genome is rapidly released into the nucleoplasm. Normally, the 153-kb HSV genome is imported by passive diffusion, but this process can be blocked by treatment with wheat germ agglutinin (WGA), which blocks nuclear pores by binding to nucleoporins containing N-acetyl-D-glucosamine (GlcNAc) residues [40], [41], [42].

MEFs were pretreated with Alexa Fluor 594-conjugated WGA (Invitrogen-Molecular probe, Carisbad, CA) that was transfected into the cells using the Chariot protein delivery system (Active Motif, Carisbad, CA). WGA was allowed to couple with the Chariot compound for 30 min at room temperature to form complexes that were incubated for 1 h with MEFs suspended in serum-free culture medium. To eliminate surface-bound WGA, cells were treated with 0.1 M GlcNAc for 10 min, according to Raub's methods [43]. The WGA-treated MEFs were then infected with MCMV at an MOI of 10. WGA nuclear pore blockage inhibited MCMV infection in a concentration-dependent manner (*P*<0.001) (Figure 10F), indicating that the number of NPCs may influence susceptibility to MCMV. Finally, the number of NPCs was measured using mAb mAb414, which recognizes 4 nucleoporins [40]. Confocal images showed that the density of nuclear pores is significantly lower in iPS and ES cells than in MEFs (*P*<0.001) (Figures 10G and 10H).

**Figure 11:**
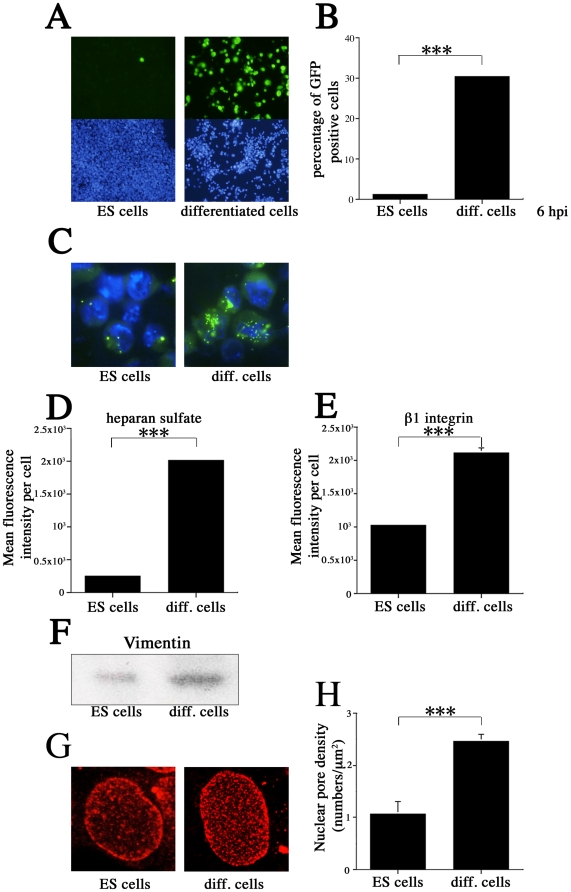
Analysis of factors for MCMV susceptibility during cell differentiation. (A) More GFP-positive cells were observed in differentiated cells than in ES cells after infection with EF-1α recombinant MCMV at an MOI of 10 at 24 hpi. (B) At 6 hpi, the proportion of GFP-positive cells after infection with EF-1α recombinant MCMV at an MOI of 10 was significantly higher in differentiated cells (30.4%) than in ES cells (1.2%) (*P*< 0.001) (C) *In situ* hybridization was used to visualize the MCMV genome in ES and differentiated cells infected with MCMV at an MOI of 10. (D) Average mean heparan sulfate fluorescence intensity per cell was 8.1-fold greater in differentiated cells than in ES cells (*P*<0.001). (E) The average mean fluorescence intensity of β1 integrin per cell is 2.1-fold greater in differentiated cells than in ES cells (*P*<0.001). (F) Western blotting revealed that per-cell expression of vimentin was significantly higher in differentiated cells than in ES cells. (G) Confocal imaging showed that the nuclear pore density is significantly higher in differentiated cells than in ES cells. (H) Densitometric analysis confirmed the results shown in (G) (*P*<0.001). All presented experiments were performed at least 3 times, and data are given as the mean±SD. ***P<0.001, *t-*test.

Next we induced differentiation of ES cells to compare the susceptibility of ES cells and differentiated cells to MCMV. ES cells on nonadherent bacterial dishes formed cellular aggregates (embryoid bodies) during growth in suspension with retinoic acid for 8 days. Embryoid bodies were dissociated and plated onto coated dishes in differentiation medium without leukemia inhibitory factor (LIF). After several passages, differentiated cells and ES cells were infected with EF-1α recombinant MCMV at an MOI of 10 and incubated for 24 hr. Differentiated cells exhibited a significantly greater proportion of GFP-positive cells than did ES cells (Figure 11A). Even at 6 hpi (prior to the onset of MCMV DNA synthesis), the proportion of GFP-positive cells after infection with EF-1α recombinant MCMV at MOI of 10 was significantly higher in differentiated cells (30.4%) than in ES cells (1.2%) (*P*<0.001) (Figure 11B). We performed ISH to observe MCMV DNA signals in ES and differentiated cells at 3 hpi (MOI of 10). The number of ISH signals is significantly higher in differentiated cells than in ES cells (Figure 11C). These results indicate that differentiated cells are more susceptible to MCMV because the MCMV genome can enter the nucleus more efficiently than in ES cells. Next, we compared several factors (heparan sulfate, β1 integrin, vimentin, nuclear pores) between ES cells and differentiated cells. Flow cytometry showed that average mean HS fluorescence intensity per cell was 8.1-fold greater in differentiated cells than in ES cells (*P*<0.001) (Figure 11D). The average mean fluorescence intensity of β1 integrin per cell was 2.1-fold greater in differentiated cells than in ES cells (*P*<0.001) (Figure 11E). Western blotting revealed that per-cell expression of vimentin was significantly higher in differentiated cells than in ES cells (Figure 11F). Confocal imaging showed that nuclear pore density was significantly higher in differentiated cells than in ES cells (*P*<0.001) (Figures 11G and 11H).

## Discussion

Previously, little was known about the susceptibility of embryonic stem cells to CMV infection. Gonczol et al. reported that infected human embryonic carcinoma cells did not express viral antigens or produce infectious virus [5]. NT2 cells, their differentiated derivatives, and MRC-5 fibroblasts take up the virus in a similar manner; at just 1 hpi, a significant fraction of applied virus is found in the nucleus of each cell type [15]. Human NT2 have long been recognized as a useful model in which to study the regulatory mechanisms behind MIE enhancer/promoter silencing during quiescent HCMV infection [5], [13], [14]. In human embryonic carcinoma cells, the MIE promoter of HCMV is activated by physiological levels of retinoic acid [44]. Treatment with TSA, a histone deacetylase (HDAC) inhibitor, rendered NT2 cells transiently permissive to HCMV, suggesting that HDACs play an important role in the repression of viral replication [18]. Treatment with FSK, an adenylyl cyclase activator, stimulates the cAMP signaling pathway, thereby alleviating MIE enhancer/promoter silencing in quiescently infected NT2 neuronal precursors. When TSA and FSK are administered simultaneously, they synergistically activate the HCMV lytic cycle. Thus, it appears that stimulation of the cAMP/protein kinase A signaling pathway drives CRE-dependent MIE enhancer/promoter activation in quiescently infected cells; this suggests one potential mode of regulation in HCMV reactivation [19].

In the current study, results of the plaque assay (Figure 2B) and IE1 protein and mRNA expression analyses (Figures 2C, 2E, and 2F) indicated that ES cells were more resistant than MEFs to MCMV, at the level of virus production. These differences seem to be a result of variations at the transcriptional or pre-transcriptional level. Since the activity of the CMV promoter in NT2 has already been the subject of intense study, we focused on the activity of the MCMV IE promoter. Activation of the integrated MCMV IE promoter was detected 2 weeks after induction of differentiation, and was predominantly observed in glial cells [4]. Here, we confirmed that the integrated MCMV IE promoter was activated in transgenic MEFs, but not in ES cells (Figures 3A and B). These results correlate with actual infectious behavior, suggesting that non-permissiveness of ES cells may depend on IE promoter activity. Thus, we hypothesized that ES may not have enough transcription factors to activate the MCMV IE promoter, or that remodeling of the chromatin associated with the IE promoter may play a pivotal role in its activation. Ishiguro et al. reported that transiently transfected DNA was rapidly assembled into a chromatinized structure in 3T3 cells, suggesting that transcription of reporter genes was at least partially repressed by chromatin organization [45]. Here, we found that integrated and transiently transfected MCMV IE promoters are activated differently. Our results showed that the transiently transfected MCMV IE promoter/enhancer has some activity in ES cells, although the MCMV IE promoter/enhancer had less activity in ES cells than in NIH3T3 cells (Figures 3C and 3D). These results indicated that ES cells contained transcriptional factors for MCMV IE promoter activation. Several reports have suggested that ES cells possess CMV promoter activity, which is compatible with our transiently transfected IE promoter results [46], [47], [48].

We found that TSA and FSK treatments significantly increased the activity of the transiently transfected MCMV IE promoter (Figure 3E and F). The integrated MCMV IE promoter responded poorly and significantly differently to TSA and FSK treatment than the transiently transfected promoter (data not shown). This likely stems from differences in promoter modification, which affect behavior [24]. Mehta et al. observed that HCMV promoter silencing is dependent on the site of transgene integration. Further, they found that the silenced CMV promoter interacts *in vivo* with methyl CpG binding protein 2 (MeCP2), a recruiter of HDACs, and histone (H3K9) methyl transferase. Histone methylation strongly correlates with the reporter expression [49]. Therefore, poor response to TSA and FSK stimulation may indicate that methyl CpG and histone methylation are important factors for silencing the integrated MCMV IE promoter in ES cells.

Unexpectedly, despite up-regulation of CREB phosphorylation, MCMV-infected ES cells exhibited a minimal responded to high-concentration FSK stimulation (Figure 4A and 4B). However, we did confirm a previous report [19] that FSK and TSA work synergistically to alleviate some, but not all, MCMV silencing (Figure 4C). Furthermore, ES cells infected with EF-1α recombinant MCMV showed very little GFP activity despite the fact that the EF-1α promoter is active in ES cells (Figure 5). Centrifugation significantly (3.9-fold) increased the proportion of GFP-positive cells in ES cultures when infected with EF-1α recombinant MCMV (Figure 6A). These results led us hypothesize that ES cells may be susceptible to MCMV infection during pre-transcription, when the virus attaches to and enters cells, and traffics to and enters the nucleus. A comparison of MCMV DNA levels in MEF and ES cells clearly showed that ES exerted multi-step inhibition that reduced MCMV DNA throughout the infection process (Figure 7). *In situ* hybridization revealed that MCMV DNA mostly localized to the nuclei of MEFs, whereas ES cells rarely had signals in this region (Figure 8). Thus, we concluded that the first barrier of resistance to MCMV infection in ES cells is a reduction of MCMV genome entry into the nucleus.

Like all herpesviruses, CMV replicates in the nucleus and requires active transfer of virions from the cell membrane to the nuclear envelope at the start of infection. CMV initiates infection via a tethering interaction between virions and cell-surface heparan sulfate proteoglycans [37]. ES cells bind approximately half as much MCMV as MEF does, although centrifugation increased susceptibility of ES cells to MCMV adsorption by 3.9-fold (Figure 6B). Additionally, ES cells have approximately 2.5-fold less HS per cell than MEFs do (Figure 9A). Nairn et al. reported that HS synthesis was enhanced 5- and 8-fold following the transition from mouse embryonic stem cell to embryoid bodies and extraembryonic endodermal cells, respectively [50]. The differentiation process may increase the amount of HS on the cell surface, while dedifferentiation from MEF to iPS may have the reverse effect.

After virions attach to heparan sulfate proteoglycans at the cell surface [37], they engage one or more receptor(s), including the integrin heterodimers α2β1, α6β1, and αvβ3 [38], [51], [52], the platelet-derived growth factor-α receptor (PDGFRα) [53], [54], and the epidermal growth factor receptor, whose role in HCMV entry is still debated [51]. In MCMV infection, the only identified entry receptor is β1 integrin [38]. In our experiment, ES cells had about 3-fold less β1 integrin per cell than did MEFs (Figure 10B), indicating that β1 integrin expression levels may control the rate of entry of MCMV into cells. We also investigated other receptor candidates, such as PDGFRα [53]. FCM analysis showed PDGFRα expression was low in both iPS and ES cells, and PDGFRα expression was not correlated to higher MCMV susceptibility. Furthermore, use of a neutralizing antibody to block PDGFRα did not decrease susceptibility to MCMV (data not shown). Based on these results, we concluded that PDGFRα does not play an important role in MCMV inhibition in ES cells.

It is also possible that MCMV uses different entry pathways in different cell types, as has been reported for different strains of HCMV infections [55], [56], [57]. In HCMV, the gH/gL/pUL (128–131A) complex is essential for infections of endothelial, epithelial, and dendritic cells, but is not needed for infection of fibroblasts and neuronal cells. HCMV gH/gL complexes are thought to play a role in promoting fusion of the viral envelope and cellular membranes, and probably act in concert with gB, potentially binding integrin receptors [38], [52], [55], [58]. Like HCMV, MCMV (Smith strain) infects many cell types, including fibroblasts, epithelial cells, endothelial cells, macrophages, and dendritic cells [59]. Mouse ES cells have shown resistance to both the Smith strain and the K181-derived RM4503 strain. As far as we know, differences in MCMV strains do not affect their ability to infect ES cells. m74 (gO)-knockout MCMV mutants enter fibroblasts by endocytosis via an energy-dependent, pH-sensitive pathway. MCMV uses 2 different pathways (fusion or endocytosis) to enter fibroblasts [60]. Although different gH/gL complexes may direct the virus to different cell types, it is not yet clear how the virus differentially uses one complex or the other. Future studies should focus on clarifying whether MCMV, like HCMV, uses an alternative gH/gL complex comparable to the gH/gL/pUL (128-131A) complex of HCMV. A combination of centrifugation and PEG treatment did not make ES cells fully permissive, like MEF. This suggests that MCMV was blocked at least one post-entry stage of infection.

Disruptions of the vimentin network greatly diminish HCMV entry into the cell nucleus. Because viral particles remain in the cytoplasm longer in vimentin-negative than in vimentin-positive cells, it has been hypothesized that viral genomes enter the nucleus via vimentin association with integrins at the cell surface, with endosomes and microtubules in the cytoplasm, and with the lamina and matrix in the nucleus [39]. This suggestion is supported by reports that ES cells express less vimentin than do differentiated cells [61], [62], [63]. Using both immunocytochemistry and western blotting, we confirmed that ES cells and iPS expressed less vimentin than did MEFs (Figure 10D). Chemical disruption of the vimentin network with acrylamide severely reduced entry of MCMV in a concentration-dependent manner (Figure 10C). Ivaska et al. reported that the efficient recycling of β1 integrins to the plasma membrane requires the PKCε-regulated phosphorylation of amino-terminal sites on vimentin. This process is also required for efficient migration on a β1 integrin substrate [64]. When vimentin expression is lacking, virions may not be capable of efficiently transferring viral capsids to the nucleus. Indeed, failure to transport particles to the nucleus and achieve nuclear genome deposition results in reductions in the cytoplasmic viral DNA levels of endothelial cells [65]. Lower levels of vimentin expression in ES cells and iPS appear to play an important role in MCMV infection by inhibiting capsid trafficking and/or docking to the nuclear envelope.

The nuclear pore is an integral part of the nuclear membrane of all eukaryotic cells. It permits the diffusion of small molecules and the active transport of larger molecules between the nucleus and cytoplasm. The mechanism by which genomes of large DNA viruses are translocated through the nuclear pore complex (NPC) are poorly understood, and appear to vary by virus species [42], [66]. Also poorly understood is the mechanism by which the CMV genome enters the nucleus; it may employ mechanisms similar to those of HSV. Yasuhara et al. showed that expression of importin-α subtypes is strictly regulated during neural differentiation in mouse ES cells, and that the switching of importin-α subtype expression is critical for neural differentiation. However, both mouse embryonic stem cells and differentiated cells constantly express importin β [67]. Future work is needed to elucidate whether these factors are important in CMV nuclear entry and, if so, what role they play.

Here, we confirmed that WGA, which is transduced into cells via the Chariot protein delivery system, inhibited MCMV infection in a concentration-dependent manner. This indicates that MCMV entered the CMV genome through the NPC and suggests that the number and/or density of functional nuclear pores may affect susceptibility to CMV by reducing the rate of capsid docking at NPCs. ES cells and iPS have lower nuclear pore densities than do MEFs, which supports previous findings that nuclear pore densities are low in ES cells, and only increase after differentiation [68]. This may explain why MCMV enters MEFs at a 2-fold higher rate than it enters ES cells. However, there may be additional mechanisms that also influence this pattern, and future work is needed to investigate this possibility.

Another aspect of this study is to look at MCMV as a gene transfer vector. Recombinant MCMV has been explored as a potential antigen delivery vector due to its ability to target human dendritic cells without compromising the antigen-presenting ability of dendritic cells[69]. Additionally, MCMV undergoes an abortive infection in human cells and is safe for use in humans [69], [70], [71]. MCMV can express transgenes and can potentially carry rather large DNA fragments since its genome is large (about 230kb) and only about 80 genes appear to be necessary for its replication. Our study indicates that recombinant MCMV may not be a suitable vector to introduce exogenous genes into pluripotent stem cells. On the other hand, there is a possibility that MCMV may introduce multiple transgenes simultaneously into differentiated human cells. Furthermore MCMV may be used to introduce exogenous genes such as Oct3/4, Sox2, Klf4, and c-myc into human fibroblasts to generate human iPS. Future work is needed to investigate this possibility.

This is the first report that the resistance of pluripotent stem cells to CMV entry is a multi-step process. In addition to the low rate of MCMV genome entry into the nucleus, it is also likely that an epigenetic inhibitory mechanism represses MCMV transcription, and that these 2 processes work together to make ES cells highly resistant to CMV infection. There may be other mechanisms to be revealed in the future. It is also probable that differentiation and dedifferentiation change the factors that we have shown to influence CMV susceptibility, including HS, β1 integrin, and vimentin expression, as well as the density of nuclear pores. We confirmed that ES cell differentiation increased susceptibility to MCMV and increased nuclear pore density and the expression of heparan sulfate, β1 integrin, and vimentin (Figure 11). These results indicate that differentiated cells are more susceptible to MCMV because the MCMV genome can enter the nucleus more efficiently than in ES cells. It has long been thought that the CMV genome enters the nuclei of pluripotent stem cells in the same manner in which it invades fibroblasts [5]. Ours is the first report that ES and iPS permit lower levels of CMV genome entry into the nucleus than MEFs do. This finding should help elucidate the relationship between CMV susceptibility and the developmental process, which may eventually lead to treatments for reducing the occurrence of congenital anomalies caused by CMV infection in humans.

## Materials and Methods

### Ethics statement

This study was carried out in strict accordance with the recommendations in the Guide for the Care and Use of Laboratory Animals of the National Institutes of Health. The protocol was approved by the Committee on the Ethics of Animal Experiments of the Hamamatsu University School of Medicine (Permit Number: 2007089). All surgery was performed under sodium pentobarbital anesthesia, and all efforts were made to minimize suffering.

### Mice

C57BL/6 mice were obtained from SLC Japan (Hamamatsu, Japan). Transgenic mouse lines that expressed the *lacZ* gene under transcriptional control of the MCMV major IE promoter 1 (MCMV IE promoter-*lacZ* transgenic) were described previously [21], [72].

### Establishment of ES Cell Lines and ES/iPS culture

ES cell lines were established as described by Matsukage et al. [4]. C57BL/6 female mice were mated with C57BL/6 or homozygous MCMV IE promoter-*lacZ* transgenic males, and the presence of vaginal plugs was verified the following morning. On gestational day (GD) 3.5, blastocysts were collected from the uterine cavity by flushing with Dulbecco's modified Eagle's medium (DMEM) supplemented with 10% FCS. Blastocysts were cultured on MEFs, which had been treated with mitomycin C, in 35-mm plastic dishes with ES/FCS medium at 37°C in a humidified atmosphere of 95% air and 5% CO_2_. The ES/FCS medium consisted of DMEM supplemented with 15% FCS, 1000 U LIF (Chemicon International, Temecula, CA), and 10^-4^ M 2-mercaptoethanol (2-ME; Sigma Chemical, St. Louis, MO), nonessential amino acids (Invitrogen Corp., Carlsbad, CA), sodium pyruvate (Invitrogen), and penicillin. The recovered blastocysts were washed with CaH- and MgH-free PBS and treated with 0.01% trypsin and 1 mM EDTA at 37°C for a few minutes. After ES/FCS medium was added to neutralize the trypsin, the inner cell mass (ICM) was dissociated into small clumps by pipetting, and the clumps were seeded into a new dish with MEF feeder cells containing ES/knockout serum replacement medium (Invitrogen), which replaced the FCS with KSR. At 5–7 days after dissociation of the ICM, small round colonies were generated in some dishes. These were trypsinized and seeded into new 35-mm dishes. Culture medium was replaced daily. Blastocysts hatched after 2 days of culture and attached to the dish surface. After 5 days the ICM of the blastocysts proliferated to form a round clump at the center of the trophoectodermal cell sheet. Nanog-iPS (APS0001, iPS-MEF-Ng-20D-17) was obtained from the RIKEN BRC Cell Bank (Tsukuba, Japan) and maintained in ES cell medium.

### Induction of ES cell differentiation into various cell types

For embryoid body formation (EB), ES cells were plated onto nonadherent bacterial dishes (Greiner) in EB medium (ES medium without LIF and only 10% FCS) and incubated for 8 days. Medium was changed every 2 days and 5 µM retinoic acid was added after 4 days. Embryoid bodies were dissociated and the cells plated. Dissociated cells were maintained with EB medium for more than 4 weeks.

### Alkaline phosphatase staining

Cells were washed twice with PBS and fixed with 4% formaldehyde (in PBS) for approximately 15 min at room temperature. The cells were washed with PBS and incubated with an alkaline phosphatase substrate solution for 10 min at room temperature. After washing with PBS, the cells were photographed.

### Immunocytochemistry

Immunocytochemical staining was performed as described previously [73]. The fixed cells (ES and MEF) on dishes were stained with anti-mouse-Nanog antibody (Reprocell, Tokyo, Japan), anti-rabbit-SOX2 antibody (Abcam, Cambridge, UK), anti-mouse-OCT3/4 antibody (Santa. Cruz Biotechnology, Santa Cruz, CA, USA) and mAb N2, specific to the MCMV IE-89K-antigen [22], and anti-mouse-vimentin (PROGEN Biotechnik GmbH, Heidelberg). The secondary antibody (green) was Alexa Fluor 488 goat anti-rabbit, -rat or -mouse IgG (H+L) (Molecular probes, Invitrogen, CA, USA), incubated for 1 h. DAPI was used to stain the cell nuclei (blue).

### Virus

The Smith strain of MCMV was provided by Dr. Y. Minamishima (Miyazaki, Japan) [74]. Recombinant MCMV (RM4503) capable of expressing EGFP was provided by Dr. Mocarski (Stanford University, Stanford, CA)[75]. RM4503 was constructed to express EGFP under control of an HCMV promoter/enhancer inserted into the MCMV ie2 gene, which has been shown to be completely dispensable for viral growth, latency, and pathogenesis in BALB/c mice [76].

### Generation of recombinant virus

Recombinant viruses, derived from the Smith strain of wild-type MCMV (gene accession number U68299) capable of expressing the EGFP (Clontech, Palo Alto, CA), were used in this study. Recombinant virus was constructed to express an EGFP gene insert under control of EF-1α (EF-1α/HTLV composite promoter that combines the EF-1α core promoter and the 5′ untranslated region of the Human T-cell Leukemia Virus. The EF-1α/HTLV promoter was taken from pSELECT plasmid (InvivoGen, San Diego, CA, USA)). EF-1α/HTLV promoter-EGFP was inserted between 184443 and 187158 in the MCMV genome by homologous recombination. This recombination causes the deletion of a 2716 nt sequence including the MCMV *ie2* gene (m128; position from 186085 to 187296), which is completely dispensable for viral growth in cell culture as well as for growth, latency, and pathogenesis in mice [76]. A recombinant virus was created by co-transfection of MEFs with MCMV Smith strain genomic DNA and a DNA fragment carrying the EF-1α/HTLV promoter-EGFP cassette with 5′-(position from 183078 to 184442) and 3′-(position from 187159 to 188573) flanking sequences using FuGENE 6 transfection reagent (Roche Diagnostics, Mannheim, Germany).

### Virus infection of ES Cells and MEFs

ES cells and MEFs were incubated with MCMV for 1.5 hr at an MOI of 1, 10, and 100 plaque-forming units per cell, washed with Hank's balanced salt solution (Invitrogen) 3 times, and cultured with fresh medium. At different times after infection, the supernatants and infected cells from 3 samples were collected. The virus was quantified by the plaque assay method of Wentworth and French [77] with MEFs, as reported previously [78]. Infected cells were processed for MCMV antigen detection by fluorescent microscopy, immunofluorescence, and flow cytometry. The numbers of GFP-positive ES cells or MEFs after infection with recombinant MCMV (RM4503) were counted in 10 high-power fields on photographs using a fluorescent microscope.

### Flow cytometry

For flow cytometry, ES cells and MEF were treated as previously described [4]. The cells were reacted in suspension with mAb N2, specific to the MCMV IE-89K-antigen. For the secondary labeling, phycoerythrin (PE)- or FITC-conjugated rabbit anti-rat immunoglobulins (Dako) were used for MCMV antigen detection. The stained cells were analyzed by flow cytometry using an EPICS profile analyzer (Coulter, Miami, FL).

### Reverse transcription-PCR (RT-PCR)

Total RNA from MEFs and ES cells was isolated at 3 hours and 6 hours after infection by using the RNeasy Mini Kit (Qiagen, Hilden Germany) according to the manufacturer's protocol. RNA samples were treated with RNAse-free DNAse I for 15 min at room temperature, and the DNAse was inactivated at 65°C for 15 min. The RNA was reverse-transcribed using oligonucleotide primers at 50°C for 50 min, and reactions were terminated by heating at 70°C for 15 min. The reverse-transcribed products were treated with RNase H for 20 min at 37°C. Quantification of the viral transcripts from genes *m123 (ie1)* was performed by real-time one-step RT-PCR with the primers and probes indicated as follows. For *ie1*-specific RT-PCR, probe ie1-taq1 directed against the exon 3/4 splicing junction comprised nucleotides 5′-6,338 to 6,328 on exon 4 and 5′-6,205 to 6,192 on exon 3 (GenBank accession no. L06816). Oligonucleotide 5′-6,393 to 6,367 served as forward primer ie1_taq_forw1, and oligonucleotide 5′-6,139 to 6,156 served as reverse primer ie1_taq_rev1, yielding an amplification product of 133 bp. The fluorogenic 5′ nuclease probe was 5′-FAM[6-carboxy-fluorescein]-AACGCTCCTCACTGCAGCATGCTTG-3′-TAMRA [6-carboxy-tetramethyl rhodamine])[79]. 18S rRNA was used to normalize for variations in RNA extraction. The 18S oligonucleotide primers were TaqMan Gene Expression assays, Endogenous control; 18S (mouse: Hs99999901_s1) (FAM). Assays were performed in triplicate using a one-step protocol consisting of an initial reverse-transcription reaction followed immediately by cDNA amplification. All TaqMan reagents were purchased from Applied Biosystems. RNA (2 µL) was added to 18 µL of PCR mix in each well of a MicroAmp optical reaction plate containing 10 µl TaqMan Fast Universal PCR Master Mix, 6.9 µL distilled water, 0.304 µL each of 50 µM ie1 forward and ie1 reverse primers, 0.492 µL of 0.26 µM FAM-labeled probe, 2 µL of a mixture of 18S forward and reverse primers, and 0.125 µL of 40 µM 18S rRNA-FAM-labeled probe. The samples were amplified in a StepOnePlus realtime PCR system (Applied Biosystems) using the following program: 1 cycle; 95°C for 20 s, 1 cycle; and 95°C for 1 s and 60°C for 20 s, 40 cycles.

### Galactosidase staining and galactosidase enzyme assay

After fixation for 1 hr in 4% paraformaldehyde (in 0.1 M PBS) at 4°C and washing with PBS, the IE promoter-*lacZ* transgenic ES or MEF cells were stained for β-gal activity using the method of Mercer et al. with X-Gal. The IE promoter-*lacZ* transgenic ES cells and transgenic MEFs and non-transgenic MEFs were fixed in 4% paraformaldehyde for 5 min and washed with PBS [80]. β-gal activity was detected by X-Gal staining, and β-gal-positive cells were photographed in 10 hpf. The β-galactosidase Enzyme Assay System (Promega) was used according to the manufacturer's protocol.

### Construction of plasmids and transfection

Plasmids were constructed from the plasmid vector pEGFP-C1 (Clontech). After the deletion of the HCMV enhancer/promoter sequence and multiple cloning sites from pEGFP-C1, PCR-amplified MCMV MIE enhancer/promoter of *ie1* and *ie3* (MCMV-MIE *pro1*) or EF-1α/HTLV composite promoter were cloned into the *Mlu*I and *Ase*I/*Nhe*I sites of pEGFP-C1, respectively. MCMV-MIE *pro1* was selected using PCR with primers specific for the *ie1* gene yielding 1338 bp. EF-1α/HTLV composite promoter combines the elongation factor 1α core promoter and the 5′ untranslated region of the Human T-cell Leukemia Virus. EF-1α/HTLV promoter was taken from the pSELECT plasmid (InvivoGen, San Diego, CA, USA). At the 5′ and 3′positions of each promoter cassette, 2 sequences with homology to the MCMV gene-containing fragment (from 183078 to 184442) to the 5′ site and another fragment (from 187159 to 188573) to the 3′site were inserted. Attractene Transfection Reagent (Qiagen, Hilden Germany) was used to transfect the plasmids into ES cells and MEFs. Transfection into ES cells and MEFs was performed using a Nucleofector electroporator (Amaxa Biosystems, Germany) according to the manufacturer’s recommended protocol.

### Drug treatment

Stock solutions of trichostatin A (1 mg/mL), sodium butylate (1400 mM), FSK (10 mM) (Sigma, St. Louis, MO) were prepared in dimethyl sulfoxide. Immediately before addition, these were diluted to the desired concentrations in DMEM.

### Western blotting

CREB protein and phosphorylated CREB protein were detected using rabbit antibody specific to CREB (Upstate Cell Signaling, Virginia) and phosphorylated CREB (Affinity BioReagents, Colorado). Sample loads were standardized by detecting β-actin using mouse mAb specific to β-actin (Sigma). The blots were incubated with biotin-conjugated secondary antibody (Nichirei, Japan) followed by horseradish peroxidase-conjugated avidin-biotin reaction. Immunoreactive bands were visualized using enhanced chemiluminescence substrate (Amersham Pharmacia Biotech, NZ).

### Centrifugal and PEG enhancement of infection

Culture dishes were centrifuged at 600 *g* for 2 h at room temperature and then moved to 37°C for 1 h. The cells were washed twice with PBS and treated with polyethylene glycol or returned to 37°C. PEG 6000 (Nakarai Tesuque Corporation, Kyoto, Japan) was prepared as a 60% (wt/wt) solution in PBS and diluted with warm PBS to 44%. Cells were treated with diluted PEG for 30 s and washed immediately 5 times with warm PBS. The treated cells were infected with EF-1α recombinant MCMV at an MOI of 50. After 24 hpi, the number of GFP-expressing cells was quantified.

### Cell collection and fractionation

Cell fractionation was performed according to Wang et al. 's protocol [33]. Cells were harvested by trypsinization and collected in 15-ml conical tubes on ice, washed 3 times with cold PBS, transferred to microfuge tubes, and pelleted at 4000 rpm for 3 min in a refrigerated centrifuge. Cell pellets were resuspended in 1 mL RSB (10 mM Tris, pH 7.4, 10 mM NaCl, 3 mM MgCl_2_), incubated for 3 min on ice, followed by centrifugation at 4°C. The volume of the swelled cell pellet was estimated and resuspended by slow pipetting with 4 volumes of lysis buffer RSBG40 [10 mM Tris, pH 7.4, 10 mM NaCl, 3 mM MgCl_2_, 10% glycerol, 0.5% Nonidet P-40, 0.5 mM dithiothreitol (DTT), and 100 U/mL rRNasin (Promega, WI)]. Nuclei were pelleted by centrifugation at 7000 rpm for 3 min, and the supernatant was recovered and saved as the cytoplasmic fraction. Nuclear pellets were resuspended in RSBG40, and one-tenth volume of detergent [3.3% (wt/wt) sodium deoxycholate and 6.6% (vol/vol) Tween 40] was added with slow vortexing, followed by incubation on ice for 5 min. Nuclei were again pelleted and the supernatant was pooled with the previous cytoplasmic fraction. Nuclear pellets were washed once more in RSBG40, collected at 10,000 rpm for 5 min, and the resulting pellet used for nuclear DNA extraction. Cell lysis and nuclear integrity was monitored by light microscopy following trypan blue staining.

### Quantification of viral genomes in cell

DNA was extracted with a DNeasy tissue kit (QIAGEN). Viral genomes were quantified by real-time PCR using the SYBR® Green PCR Master Mix (Applied Biosystems). A 2 µl aliquot of the DNA was added as template DNA to a reaction mixture that included the 2X SYBR® Green PCR Master Mix with 1 µM of each primer. A fragment of the *gB* gene was amplified from mCMV virion DNA by PCR using oligonucleotides gB-forw (5′-GAAGATCCGCATGTCCTTCAG-3′) and gB-rev (5′-AATCCGTCCAACATCTTGTCG-3′) [81], and primers for amplification of a fragment of the cellular β-actin gene were forward, (5′-GACGGCCAAGTCATCACTATTG-3′), reverse, (5′-AGGAAGGCTGGAAAAGAGCC-3’). PCR was performed with the following cycling conditions. The samples were amplified in a StepOnePlus realtime PCR system (Applied Biosystems): 95°C for 10 min, 1 cycle; and 95°C for 15 s and 60°C for 60 s, 40 cycles. Semiquantitative PCRs were performed under the following conditions with using the Phusion kit (FINNZYMES, Espoo, Finland): 1 cycle at 94°C for 30 s; 30 cycles of 10 s at 94°C, 10 s at the corresponding annealing temperature, and 20 s at 72°C; and 1 cycle at 72°C for 5 min.

### *In situ* hybridization

The probe for DNA *in situ* hybridization, pSM3fr, was made from the MCMV DNA genome with a bacterial artificial chromosome system by nick translation as described previously [34]. *In situ* hybridization to MCMV DNA was performed as described previously [34]. The cells were treated with RNase (Boehringer; 100 µg/mL in PBS) for detection of viral DNA.

### Nuclear pore inhibition with wheat germ agglutinin

Wheat germ agglutinin was allowed to couple with the Chariot compound (Active Motif, Carisbad, CA) for 30 min at room temperature to form complexes. The complexes were incubated for 1 h with MEFs suspended in serum-free culture medium. Successful transfection of WGA into MEFs results in blockage of the nuclear pores. To eliminate surface-bound WGA, cells were treated with 0.1 M GlcNAc for 10 min, according to Raub's methods [43].

## References

[1] M H (1991). Congenital and perinatal human cytomegalovirus infection..

[2] Kawasaki H, Kosugi I, Arai Y, Tsutsui Y (2002). The amount of immature glial cells in organotypic brain slices determines the susceptibility to murine cytomegalovirus infection.. Lab Invest.

[3] Kawasaki H, Mocarski ES, Kosugi I, Tsutsui Y (2007). Cyclosporine inhibits mouse cytomegalovirus infection via a cyclophilin-dependent pathway specifically in neural stem/progenitor cells.. J Virol.

[4] Matsukage S, Kosugi I, Kawasaki H, Miura K, Kitani H (2006). Mouse embryonic stem cells are not susceptible to cytomegalovirus but acquire susceptibility during differentiation.. Birth Defects Res A Clin Mol Teratol.

[5] Gonczol E, Andrews PW, Plotkin SA (1984). Cytomegalovirus replicates in differentiated but not in undifferentiated human embryonal carcinoma cells.. Science.

[6] Reeves MB, Lehner PJ, Sissons JG, Sinclair JH (2005). An in vitro model for the regulation of human cytomegalovirus latency and reactivation in dendritic cells by chromatin remodelling.. J Gen Virol.

[7] Odeberg J, Wolmer N, Falci S, Westgren M, Seiger A (2006). Human cytomegalovirus inhibits neuronal differentiation and induces apoptosis in human neural precursor cells.. J Virol.

[8] Tsutsui Y (2009). Effects of cytomegalovirus infection on embryogenesis and brain development.. Congenit Anom (Kyoto).

[9] Krmpotic A, Bubic I, Polic B, Lucin P, Jonjic S (2003). Pathogenesis of murine cytomegalovirus infection.. Microbes Infect.

[10] Tsutsui Y, Kosugi I, Kawasaki H (2005). Neuropathogenesis in cytomegalovirus infection: indication of the mechanisms using mouse models.. Rev Med Virol.

[11] Schleiss MR (2002). Animal models of congenital cytomegalovirus infection: an overview of progress in the characterization of guinea pig cytomegalovirus (GPCMV).. J Clin Virol.

[12] Kashiwai A, Kawamura N, Kadota C, Tsutsui Y (1992). Susceptibility of mouse embryo to murine cytomegalovirus infection in early and mid-gestation stages.. Arch Virol.

[13] LaFemina R, Hayward GS (1986). Constitutive and retinoic acid-inducible expression of cytomegalovirus immediate-early genes in human teratocarcinoma cells.. J Virol.

[14] Meier JL, Stinski MF (1997). Effect of a modulator deletion on transcription of the human cytomegalovirus major immediate-early genes in infected undifferentiated and differentiated cells.. J Virol.

[15] Gonczol E, Andrews PW, Plotkin SA (1985). Cytomegalovirus infection of human teratocarcinoma cells in culture.. J Gen Virol.

[16] Nelson JA, Groudine M (1986). Transcriptional regulation of the human cytomegalovirus major immediate-early gene is associated with induction of DNase I-hypersensitive sites.. Mol Cell Biol.

[17] Meier JL (2001). Reactivation of the human cytomegalovirus major immediate-early regulatory region and viral replication in embryonal NTera2 cells: role of trichostatin A, retinoic acid, and deletion of the 21-base-pair repeats and modulator.. J Virol.

[18] Murphy JC, Fischle W, Verdin E, Sinclair JH (2002). Control of cytomegalovirus lytic gene expression by histone acetylation.. Embo J.

[19] Keller MJ, Wu AW, Andrews JI, McGonagill PW, Tibesar EE (2007). Reversal of human cytomegalovirus major immediate-early enhancer/promoter silencing in quiescently infected cells via the cyclic AMP signaling pathway.. J Virol.

[20] Mocarski E, Shenk T, Pass R, Knipe DM, Howley PM (2007). Cytomegaloviruses.. Virology. 5th ed.

[21] Aiba-Masago S, Baba S, Li RY, Shinmura Y, Kosugi I (1999). Murine cytomegalovirus immediate-early promoter directs astrocyte-specific expression in transgenic mice.. Am J Pathol.

[22] Shinmura Y, Aiba-Masago S, Kosugi I, Li RY, Baba S (1997). Differential expression of the immediate-early and early antigens in neuronal and glial cells of developing mouse brains infected with murine cytomegalovirus.. Am J Pathol.

[23] Stenberg RM, Fortney J, Barlow SW, Magrane BP, Nelson JA (1990). Promoter-specific trans activation and repression by human cytomegalovirus immediate-early proteins involves common and unique protein domains.. J Virol.

[24] Smith CL, Hager GL (1997). Transcriptional regulation of mammalian genes in vivo. A tale of two templates.. J Biol Chem.

[25] Montminy M (1997). Transcriptional regulation by cyclic AMP.. Annu Rev Biochem.

[26] Shaywitz AJ, Greenberg ME (1999). CREB: a stimulus-induced transcription factor activated by a diverse array of extracellular signals.. Annu Rev Biochem.

[27] Kawabata K, Sakurai F, Yamaguchi T, Hayakawa T, Mizuguchi H (2005). Efficient gene transfer into mouse embryonic stem cells with adenovirus vectors.. Mol Ther.

[28] Huber MT, Tomazin R, Wisner T, Boname J, Johnson DC (2002). Human cytomegalovirus US7, US8, US9, and US10 are cytoplasmic glycoproteins, not found at cell surfaces, and US9 does not mediate cell-to-cell spread.. J Virol.

[29] Miller N, Hutt-Fletcher LM (1992). Epstein-Barr virus enters B cells and epithelial cells by different routes.. J Virol.

[30] Nicola AV, McEvoy AM, Straus SE (2003). Roles for endocytosis and low pH in herpes simplex virus entry into HeLa and Chinese hamster ovary cells.. J Virol.

[31] Lentz BR, Lee JK (1999). Poly(ethylene glycol) (PEG)-mediated fusion between pure lipid bilayers: a mechanism in common with viral fusion and secretory vesicle release?. Mol Membr Biol.

[32] Ligas MW, Johnson DC (1988). A herpes simplex virus mutant in which glycoprotein D sequences are replaced by beta-galactosidase sequences binds to but is unable to penetrate into cells.. J Virol.

[33] Wang Y, Zhu W, Levy DE (2006). Nuclear and cytoplasmic mRNA quantification by SYBR green based real-time RT-PCR.. Methods.

[34] Tang Q, Bell P, Tegtmeyer P, Maul GG (2000). Replication but not transcription of simian virus 40 DNA is dependent on nuclear domain 10.. J Virol.

[35] Takahashi K, Yamanaka S (2006). Induction of pluripotent stem cells from mouse embryonic and adult fibroblast cultures by defined factors.. Cell.

[36] Okita K, Ichisaka T, Yamanaka S (2007). Generation of germline-competent induced pluripotent stem cells.. Nature.

[37] Compton T, Nowlin DM, Cooper NR (1993). Initiation of human cytomegalovirus infection requires initial interaction with cell surface heparan sulfate.. Virology.

[38] Feire AL, Koss H, Compton T (2004). Cellular integrins function as entry receptors for human cytomegalovirus via a highly conserved disintegrin-like domain.. Proc Natl Acad Sci U S A.

[39] Miller MS, Hertel L (2009). Onset of human cytomegalovirus replication in fibroblasts requires the presence of an intact vimentin cytoskeleton.. J Virol.

[40] Davis LI, Blobel G (1987). Nuclear pore complex contains a family of glycoproteins that includes p62: glycosylation through a previously unidentified cellular pathway.. Proc Natl Acad Sci U S A.

[41] Finlay DR, Meier E, Bradley P, Horecka J, Forbes DJ (1991). A complex of nuclear pore proteins required for pore function.. J Cell Biol.

[42] Ojala PM, Sodeik B, Ebersold MW, Kutay U, Helenius A (2000). Herpes simplex virus type 1 entry into host cells: reconstitution of capsid binding and uncoating at the nuclear pore complex in vitro.. Mol Cell Biol.

[43] Raub TJ, Koroly MJ, Roberts RM (1990). Rapid endocytosis and recycling of wheat germ agglutinin binding sites on CHO cells: evidence for two compartments in a nondegradative pathway.. J Cell Physiol.

[44] Ghazal P, DeMattei C, Giulietti E, Kliewer SA, Umesono K (1992). Retinoic acid receptors initiate induction of the cytomegalovirus enhancer in embryonal cells.. Proc Natl Acad Sci U S A.

[45] Ishiguro K, Sartorelli AC (2004). Activation of transiently transfected reporter genes in 3T3 Swiss cells by the inducers of differentiation/apoptosis—dimethylsulfoxide, hexamethylene bisacetamide and trichostatin A.. Eur J Biochem.

[46] Wang R, Liang J, Jiang H, Qin LJ, Yang HT (2008). Promoter-dependent EGFP expression during embryonic stem cell propagation and differentiation.. Stem Cells Dev.

[47] Kim S, Kim GJ, Miyoshi H, Moon SH, Ahn SE (2007). Efficiency of the elongation factor-1alpha promoter in mammalian embryonic stem cells using lentiviral gene delivery systems.. Stem Cells Dev.

[48] Hong S, Hwang DY, Yoon S, Isacson O, Ramezani A (2007). Functional analysis of various promoters in lentiviral vectors at different stages of in vitro differentiation of mouse embryonic stem cells.. Mol Ther.

[49] Mehta AK, Majumdar SS, Alam P, Gulati N, Brahmachari V (2009). Epigenetic regulation of cytomegalovirus major immediate-early promoter activity in transgenic mice.. Gene.

[50] Nairn AV, Kinoshita-Toyoda A, Toyoda H, Xie J, Harris K (2007). Glycomics of proteoglycan biosynthesis in murine embryonic stem cell differentiation.. J Proteome Res.

[51] Isaacson MK, Feire AL, Compton T (2007). Epidermal growth factor receptor is not required for human cytomegalovirus entry or signaling.. J Virol.

[52] Wang X, Huang DY, Huong SM, Huang ES (2005). Integrin alphavbeta3 is a coreceptor for human cytomegalovirus.. Nat Med.

[53] Soroceanu L, Akhavan A, Cobbs CS (2008). Platelet-derived growth factor-alpha receptor activation is required for human cytomegalovirus infection.. Nature.

[54] Wang X, Huong SM, Chiu ML, Raab-Traub N, Huang ES (2003). Epidermal growth factor receptor is a cellular receptor for human cytomegalovirus.. Nature.

[55] Patrone M, Secchi M, Bonaparte E, Milanesi G, Gallina A (2007). Cytomegalovirus UL131-128 products promote gB conformational transition and gB-gH interaction during entry into endothelial cells.. J Virol.

[56] Sinzger C (2008). Entry route of HCMV into endothelial cells.. J Clin Virol.

[57] Wang D, Yu QC, Schroer J, Murphy E, Shenk T (2007). Human cytomegalovirus uses two distinct pathways to enter retinal pigmented epithelial cells.. Proc Natl Acad Sci U S A.

[58] Vanarsdall AL, Ryckman BJ, Chase MC, Johnson DC (2008). Human cytomegalovirus glycoproteins gB and gH/gL mediate epithelial cell-cell fusion when expressed either in cis or in trans.. J Virol.

[59] Hsu KM, Pratt JR, Akers WJ, Achilefu SI, Yokoyama WM (2009). Murine cytomegalovirus displays selective infection of cells within hours after systemic administration.. J Gen Virol.

[60] Scrivano L, Esterlechner J, Muhlbach H, Ettischer N, Hagen C (2010). The m74 gene product of murine cytomegalovirus (MCMV) is a functional homolog of human CMV gO and determines the entry pathway of MCMV.. J Virol.

[61] Eastham AM, Spencer H, Soncin F, Ritson S, Merry CL (2007). Epithelial-mesenchymal transition events during human embryonic stem cell differentiation.. Cancer Res.

[62] Coonen E, Dumoulin JC, Ramaekers FC (1993). Intermediate filament protein expression in early developmental stages of the mouse. A confocal scanning laser microscopy study of in vitro fertilized and in vitro cultured pre-implantation mouse embryos.. Histochemistry.

[63] Bouhon IA, Joannides A, Kato H, Chandran S, Allen ND (2006). Embryonic stem cell-derived neural progenitors display temporal restriction to neural patterning.. Stem Cells.

[64] Ivaska J, Vuoriluoto K, Huovinen T, Izawa I, Inagaki M (2005). PKCepsilon-mediated phosphorylation of vimentin controls integrin recycling and motility.. Embo J.

[65] Slobbe-van Drunen ME, Hendrickx AT, Vossen RC, Speel EJ, van Dam-Mieras MC (1998). Nuclear import as a barrier to infection of human umbilical vein endothelial cells by human cytomegalovirus strain AD169.. Virus Res.

[66] Harel A, Forbes DJ (2001). Welcome to the nucleus: CAN I take your coat?. Nat Cell Biol.

[67] Yasuhara N, Shibazaki N, Tanaka S, Nagai M, Kamikawa Y (2007). Triggering neural differentiation of ES cells by subtype switching of importin-alpha.. Nat Cell Biol.

[68] Perez-Terzic C, Faustino RS, Boorsma BJ, Arrell DK, Niederlander NJ (2007). Stem cells transform into a cardiac phenotype with remodeling of the nuclear transport machinery.. Nat Clin Pract Cardiovasc Med.

[69] Wang X, Messerle M, Sapinoro R, Santos K, Hocknell PK (2003). Murine cytomegalovirus abortively infects human dendritic cells, leading to expression and presentation of virally vectored genes.. J Virol.

[70] Jurak I, Brune W (2006). Induction of apoptosis limits cytomegalovirus cross-species infection.. Embo J.

[71] Tang Q, Maul GG (2006). Mouse cytomegalovirus crosses the species barrier with help from a few human cytomegalovirus proteins.. J Virol.

[72] Li RY, Baba S, Kosugi I, Arai Y, Kawasaki H (2001). Activation of murine cytomegalovirus immediate-early promoter in cerebral ventricular zone and glial progenitor cells in transgenic mice.. Glia.

[73] Kosugi I, Shinmura Y, Kawasaki H, Arai Y, Li RY (2000). Cytomegalovirus infection of the central nervous system stem cells from mouse embryo: a model for developmental brain disorders induced by cytomegalovirus.. Lab Invest.

[74] Ebihara K, Minamishima Y (1984). Protective effect of biological response modifiers on murine cytomegalovirus infection.. J Virol.

[75] van Den Pol AN, Mocarski E, Saederup N, Vieira J, Meier TJ (1999). Cytomegalovirus cell tropism, replication, and gene transfer in brain.. J Neurosci.

[76] Cardin RD, Abenes GB, Stoddart CA, Mocarski ES (1995). Murine cytomegalovirus IE2, an activator of gene expression, is dispensable for growth and latency in mice.. Virology.

[77] Wentworth BB, French L (1970). Plaque assay of cytomegalovirus strains of human origin.. Proc Soc Exp Biol Med.

[78] Tsutsui Y (1995). Developmental disorders of the mouse brain induced by murine cytomegalovirus: animal models for congenital cytomegalovirus infection.. Pathol Int.

[79] Simon CO, Holtappels R, Tervo HM, Bohm V, Daubner T (2006). CD8 T cells control cytomegalovirus latency by epitope-specific sensing of transcriptional reactivation.. J Virol.

[80] Mercer EH, Hoyle GW, Kapur RP, Brinster RL, Palmiter RD (1991). The dopamine beta-hydroxylase gene promoter directs expression of E. coli lacZ to sympathetic and other neurons in adult transgenic mice.. Neuron.

[81] Simon CO, Seckert CK, Dreis D, Reddehase MJ, Grzimek NK (2005). Role for tumor necrosis factor alpha in murine cytomegalovirus transcriptional reactivation in latently infected lungs.. J Virol.

